# T cell expression of IL-18R and DR3 is essential for non-cognate stimulation of Th1 cells and optimal clearance of intracellular bacteria

**DOI:** 10.1371/journal.ppat.1006566

**Published:** 2017-08-17

**Authors:** Oanh H. Pham, Hope O’Donnell, Aymen Al-Shamkhani, Tobias Kerrinnes, Renée M. Tsolis, Stephen J. McSorley

**Affiliations:** 1 Center for Comparative Medicine and Department of Anatomy, Physiology and Cell Biology, School of Veterinary Medicine, University of California Davis, Davis, California, United States of America; 2 Faculty of Medicine, University of Southampton, Southampton, United Kingdom; 3 Department of Medical Microbiology and Immunology, School of Medicine, University of California at Davis, Davis, California, United States of America; University of Illinois, UNITED STATES

## Abstract

Th1 cells can be activated by TCR-independent stimuli, but the importance of this pathway in vivo and the precise mechanisms involved require further investigation. Here, we used a simple model of non-cognate Th1 cell stimulation in *Salmonella*-infected mice to examine these issues. CD4 Th1 cell expression of both IL-18R and DR3 was required for optimal IFN-γ induction in response to non-cognate stimulation, while IL-15R expression was dispensable. Interestingly, effector Th1 cells generated by immunization rather than live infection had lower non-cognate activity despite comparable IL-18R and DR3 expression. Mice lacking T cell intrinsic expression of MyD88, an important adapter molecule in non-cognate T cell stimulation, exhibited higher bacterial burdens upon infection with *Salmonella*, *Chlamydia* or *Brucella*, suggesting that non-cognate Th1 stimulation is a critical element of efficient bacterial clearance. Thus, IL-18R and DR3 are critical players in non-cognate stimulation of Th1 cells and this response plays an important role in protection against intracellular bacteria.

## Author summary

CD4 Th1 cells utilize a highly specific T cell receptor to identify infected cells and initiate pathogen killing via IFN-γ secretion. An alternative pathway of Th1 cell activation can occur in the absence of specific pathogen recognition, but less is known about the signals involved and the overall importance of this pathway. Here, we show that two key mediators, IL-18 and TL1A are essential for initiating alternative Th1 cell stimulation and that this pathway is essential for rapid clearance of three important bacterial infections.

## Introduction

Naïve CD4 T cells express surface T cell receptors (TCRs) that identify foreign antigenic peptides bound to major histocompatibility complex (MHC) molecules on the surface of lymph node dendritic cells [1]. Signaling via the TCR induces these naïve T cells to proliferate rapidly and integrate additional signals that allow them to differentiate into Th1, Th2, Th17, or TFH effector phenotypes [2,3]. These different effector subsets perform a variety of specialized protective functions to combat a wide range of potential pathogens that could infect any given host. In particular, Th1 cells gain the capacity to secrete IFN-γ, a key cytokine involved in the activation of macrophages and host protection against intracellular pathogens [4,5]

Effector CD4 cells can secrete cytokines after recognition of peptide/MHC complexes displayed on tissue antigen presenting cells [6,7], thus targeting inflammatory cytokine production locally to the precise anatomical site of infection. However, IFN-γ elicited from Th1 cells can also activate neighboring macrophages [8], providing a degree of non-specific defense in the immediate vicinity of Th1 stimulation. Furthermore, Th1 cells can also respond to non-TCR (non-cognate) stimuli, allowing cytokine secretion in response to local inflammatory cues, rather than antigen recognition [9]. Indeed, many previous studies have demonstrated that antigen-experienced CD8 T cells secrete IFN-γ in response to non-cognate stimuli in the context of infection [10–12]. The major signals driving CD8 non-cognate stimulation appear to be IL-12 and IL-18 [13–15], however, a much wider range of cytokines can also participate in this process [16]. Non-cognate stimulation of CD4 T cells is less well understood, but Th1, Th2, and Th17 cells all have some capability to be stimulated upon encounter with a variety of cytokines [17], implying that this could be a common feature of the response to infection. Previous studies in *Salmonella*-infected mice, where Th1 cells are the major effector subset [18], show that IL-12 and IL-18 play a critical role driving non-cognate Th1 cell stimulation [19,20]. However, IL-12 and IL-18 alone are insufficient to drive maximal IFN-γ production from Th1 cells, and the importance of other non-cognate signals is poorly understood. Furthermore, the overall contribution of cognate versus non-cognate CD4 stimulation is incompletely defined in vivo.

Death receptor 3 (DR3) (also known as TNFRSF25, TRAMP, LARD, or WSL-1) is a death-domain-containing tumor necrosis factor (TNF)-family receptor that is preferentially expressed by antigen-experienced lymphocytes and regulates lymphocyte apoptosis [21–25]. TL1A (also known as TNFSF15, VEGI) is a cytokine of the TNF family that induces signals via DR3. In humans, TL1A can also bind a decoy receptor TR6/DcR3, which is thought to function as a negative regulator of TL1A/DR3 signaling [26]. Initial experiments found that endothelial cells produced TL1A after exposure to TNF-α and IL-1 [27], however, TL1A is also produced by macrophages and dendritic cells following stimulation with immune complexes or exposure to bacterial ligands [28–30]. TL1A was initially thought to provide a co-stimulatory signal that enhances T cell proliferation and the production of IL-2 and IFN-γ [27]. Importantly, TL1A can also synergize with IL-12 and IL-18 to significantly enhance IFN-γ production [31–33], suggesting a potential role for this cytokine in non-cognate T cell responses. Indeed, a population of human memory CD4 T cells expressing IL-18Rα and DR3 has been described that produces multiple cytokines if exposed to a cocktail of IL-12, IL-18, IL-15, and TL1A [34]. The ability of TL1A to augment cytokine production in these studies suggests that TL1A/DR3 could be an important driver of inflammatory pathology [31,35]. However, recent studies show that DR3-, and TL1A-deficient mice develop severe intestinal pathology after DSS administration [36], suggesting a protective role for TL1A/DR3 in chronic inflammatory diseases. Fewer studies have examined the role of DR3 and TL1A in infectious disease models, but it is known that DR3-deficient mice display slower clearance of *Salmonella* infection and enhanced susceptibility to viral infections [30,36,37], suggesting that TL1A could be critical to effective host defense.

In this study, we examined the mechanism of non-cognate Th1 cell stimulation in *Salmonella*-infected mice and explored the contribution of this pathway to protection against a variety of intracellular pathogens. The data show that T cell-intrinsic expression of IL-18R and DR3 is required for optimal IFN-γ induction by Th1 cells in response to non-cognate stimulation, while IL-15R expression is dispensable. Effector Th1 cells generated by immunization rather than infection also have a lower capacity for non-cognate activity, despite comparable IL-18R and DR3 expression on Th1 cells. Mice lacking T cell intrinsic expression of MyD88, an important adapter molecule in non-cognate T cell stimulation, exhibit higher bacterial burdens after infection with *Salmonella*, *Chlamydia* or *Brucella*. Overall, these data show that both IL-18R and DR3 are critical for non-cognate stimulation of Th1 cells and that this response is important for protection against intracellular bacteria.

## Results

### CD4 Th1 cells in infected mice respond rapidly to non-cognate stimuli

**Fig 1:**
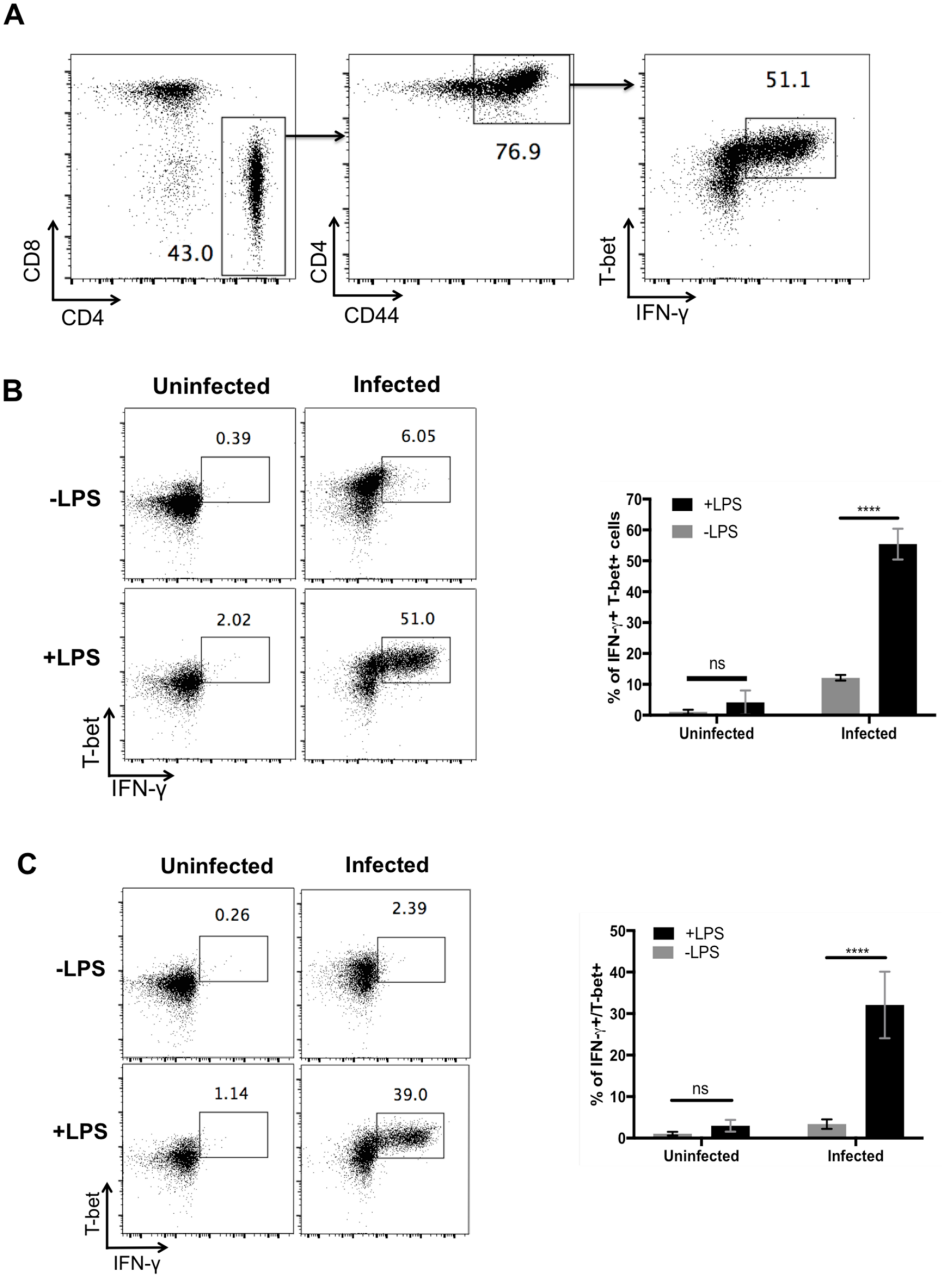
LPS induces Th1 cell IFN-γ production. C57BL/6 mice were infected intravenously (i.v.) with 5×10^5^ *Salmonella* Typhimurium or 1x10^7^ *Chlamydia muridarum*. Two weeks *(Salmonella)*, or one week *(Chlamydia)* later, infected or uninfected mice were injected i.v. with 10ug LPS, and spleens were collected 4 hours later to determine IFN-γ production. (A) Gating strategy used for examining IFN-γ production by Th1 cells. Splenocytes were gated as live, singlet, and activated CD4^+^CD8^-^ cells. This cell population was then examined for IFN-γ production and T-bet expression. Representative flow cytometry plots and a graph from combined data are shown for (B) *Salmonella*-infected mice (B) and (C) *Chlamydia*-infected mice. Data shown are combined from two experiments containing at least three mice per group. Statistical analyses were performed using two-way ANOVA with a Bonferroni post test. Error bars shown represent mean ±SEM with p < 0.0001 (****), 0.0002 (***), 0.0021 (**), 0.0332 (*), 0.1234 (ns).

Th1 cells can be activated by cognate or non-cognate stimuli to secrete IFN-γ, a cytokine critical for the resolution of intracellular bacterial infections [9]. It has been previously demonstrated that injection of ultrapure *E*. *coli* LPS initiates non-cognate T cell activity from *Salmonella*-specific CD4 T cells [19,20]. As expected, the majority of CD44^Hi^ CD4 T cells in the spleen of *Salmonella*-infected mice expressed the transcription factor T-bet and produced IFN-γ rapidly within 4 hours of LPS injection (Fig 1A). The production of IFN-γ was low from Th1 cells in uninfected mice and even in *Salmonella*-infected mice before LPS injection (Fig 1B). This time point represents peak bacterial load and therefore the small proportion of Th1 IFN-γ production in the absence of LPS does not reflect a weak response to low bacterial load. However, within hours of LPS stimulation the fraction of CD44^Hi^/T-bet^+^ cells secreting IFN-γ rose to over 50% (Fig 1B), demonstrating the robust capacity of these Th1 cells to secrete cytokine. Similar rapid responsiveness of Th1 cells was observed after LPS stimulation in *Chlamydia*-infected mice (Fig 1C), although the proportion of IFN-γ producing Th1 cells was slightly lower. Thus, Th1 cells generated in response to intracellular bacterial infection acquire the capability to respond rapidly to non-cognate stimuli.

### LPS-driven inflammation induces a variety of cytokines in infected mice

**Fig 2:**
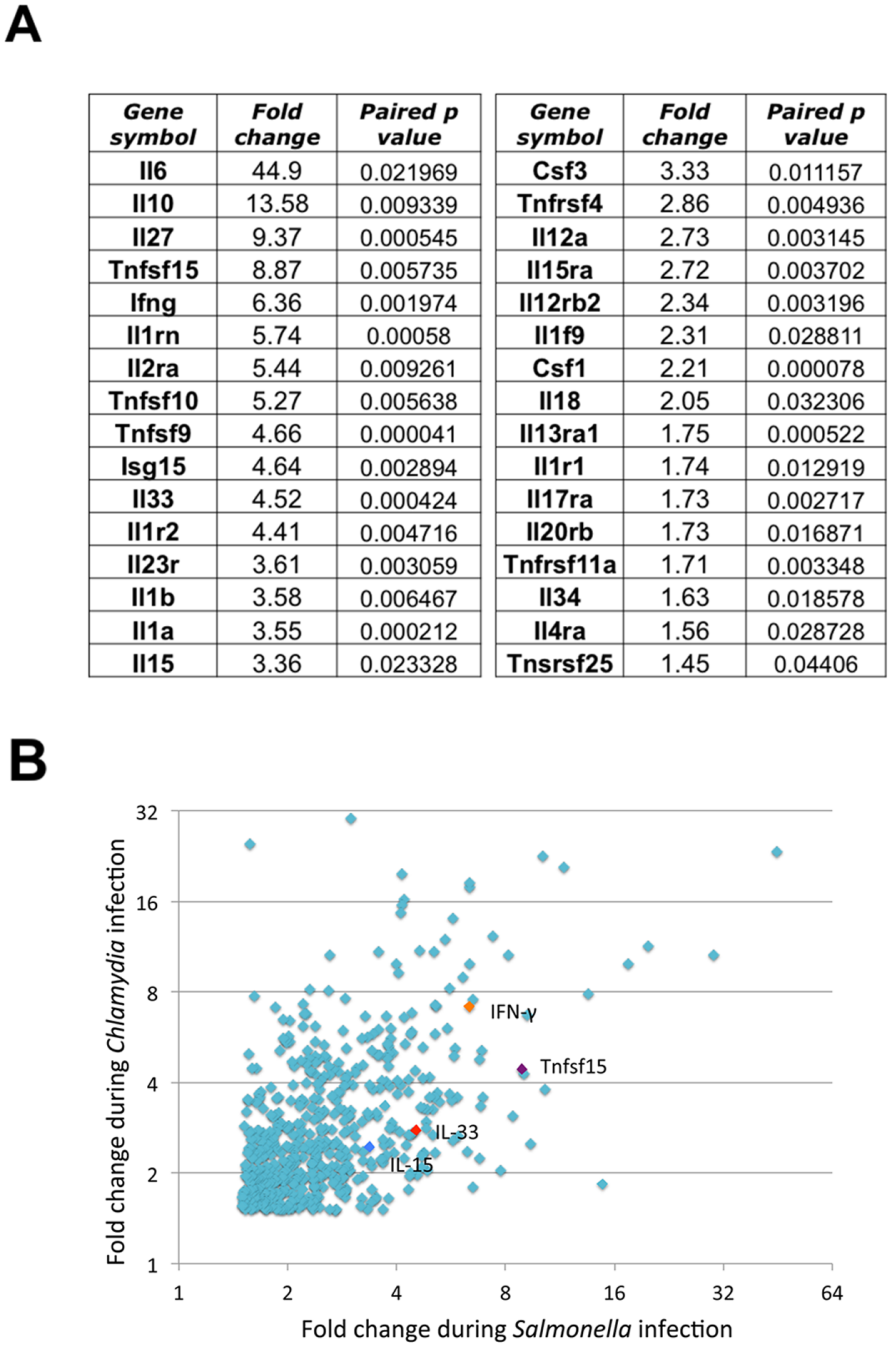
LPS induces a variety of cytokines in infected mice. C57BL/6 mice were infected intravenously (i.v.) with 5×10^5^ *Salmonella* Typhimurium or 1x10^7^ *Chlamydia muridarum*. Two weeks *(Salmonella)* or one week *(Chlamydia)* later, infected mice were injected i.v. with 10μg LPS or untreated, before spleens were collected 4 hours later, and snap frozen prior to total RNA extraction for microarray analysis using the Affymetrix GeneChip Mouse Gene 1.0 ST Array. Data were analyzed in dchip to provide mean gene expression values and fold-change comparisons between LPS-treated and untreated group means for each infection. (A) A select list of genes of interest for which RNA expression was upregulated in the spleen of *Salmonella*-infected mice 4 hours after LPS stimulation. Means were compared by paired t-test to generate the indicated p values. (B) Scatter plot shows the upregulation of genes that occur in both *Salmonella*-infected (x-axis) and *Chlamydia*-infected (y-axis) mice after LPS stimulation, with genes of particular interest highlighted. Microarray results were filtered to include only genes upregulated at least 1.5-fold, with p values greater than 0.05, for both datasets. Data were generated from 3 mice per experimental group. Data analyses, statistics, and filtering were performed in dchip, comparison of datasets, sorting, and scatterplot used Microsoft Excel.

**Fig 3:**
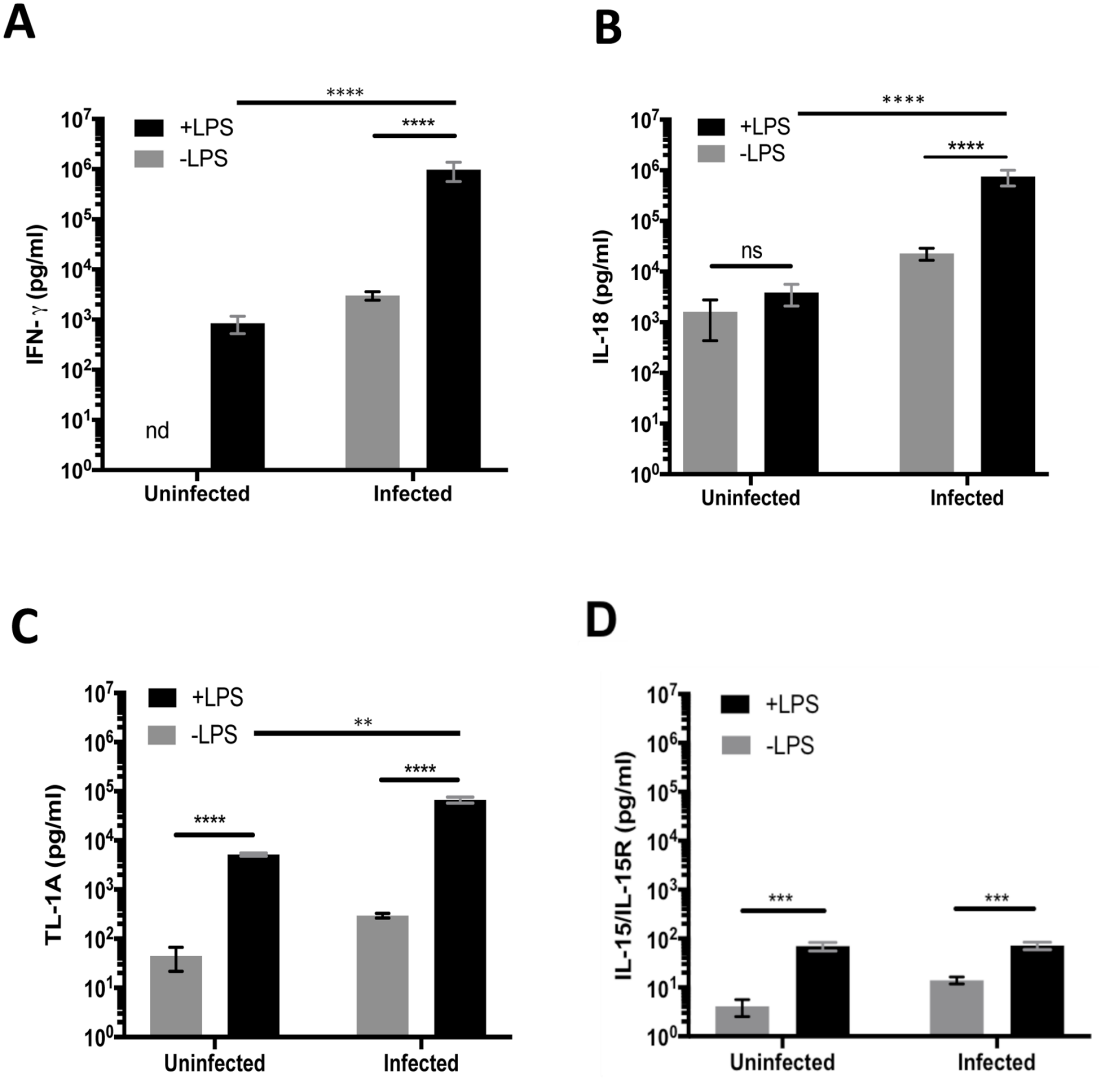
LPS induces elevated serum cytokines. C57BL/6 mice were infected intravenously (i.v.) with 5×10^5^ *Salmonella* Typhimurium, and two weeks later, infected or uninfected mice were injected (i.v.) with LPS. Serum was collected 4 hours later for determining cytokine concentration by ELISA. Graphs show concentration of serum IFN-γ (A), IL-18 (B), TL1A (C), and IL-15 (D). Data shown were combined from two experiments containing at least three mice per group (nd = none detected). Statistical analyses were performed by two-way ANOVA with a Bonferroni posttest. Error bars represent the mean ±SEM. p < 0.0001 (****), 0.0002 (***), 0.0021 (**), 0.0332 (*), 0.1234 (ns)

Although T cells can express surface Toll-like Receptors (TLRs) [38], Th1 cells in *Salmonella*-infected mice do not respond directly to LPS, but instead respond to LPS-driven inflammatory mediators [20]. Levels of mRNA for multiple cytokines and cytokine receptors were increased in the spleen of *Salmonella*-infected mice following LPS administration (Fig 2A), and many of these responses were also detected in *Chlamydia*-infected mice (Fig 2B). Several of these mediators induced by LPS administration have previously been reported to induce IFN-γ production from CD4 or CD8 T cells, including, IL-1, IL-10, IL-12, IL-15, IL-18, IL-27, IL-33, and ISG15, although some can also inhibit IFN-γ production, depending on the presence of other cytokines [9,13,16,34,39]. Since human memory CD4 T cells have recently been shown to respond synergistically to IL-15, IL-18, and TL1A [34], we decided to focus on these three potential mediators of non-cognate IFN-γ production. IFN-γ, IL-18, TL1A, and IL-15/IL-15R production were each detected in the serum of uninfected C57BL/6 mice after LPS injection (Fig 3, Uninfected+LPS). However, injection of *Salmonella*-infected mice with LPS induced much higher serum concentrations of IFN-γ, IL-18, and TL1A compared to uninfected mice or infected mice without LPS (Fig 3A–3C). In contrast, circulating IL-15/IL-15R increased similarly in response to LPS in both uninfected and *Salmonella*-infected mice (Fig 3D). In summary, the injection of LPS leads to a large increase in circulating IL-18, TL1A, and IL-15 in *Salmonella*-infected mice, and this pattern of cytokine production is in broad agreement with microarray data.

### Th1 cell expression of IL-18R and DR3 is required for optimal IFN-γ production

**Fig 4:**
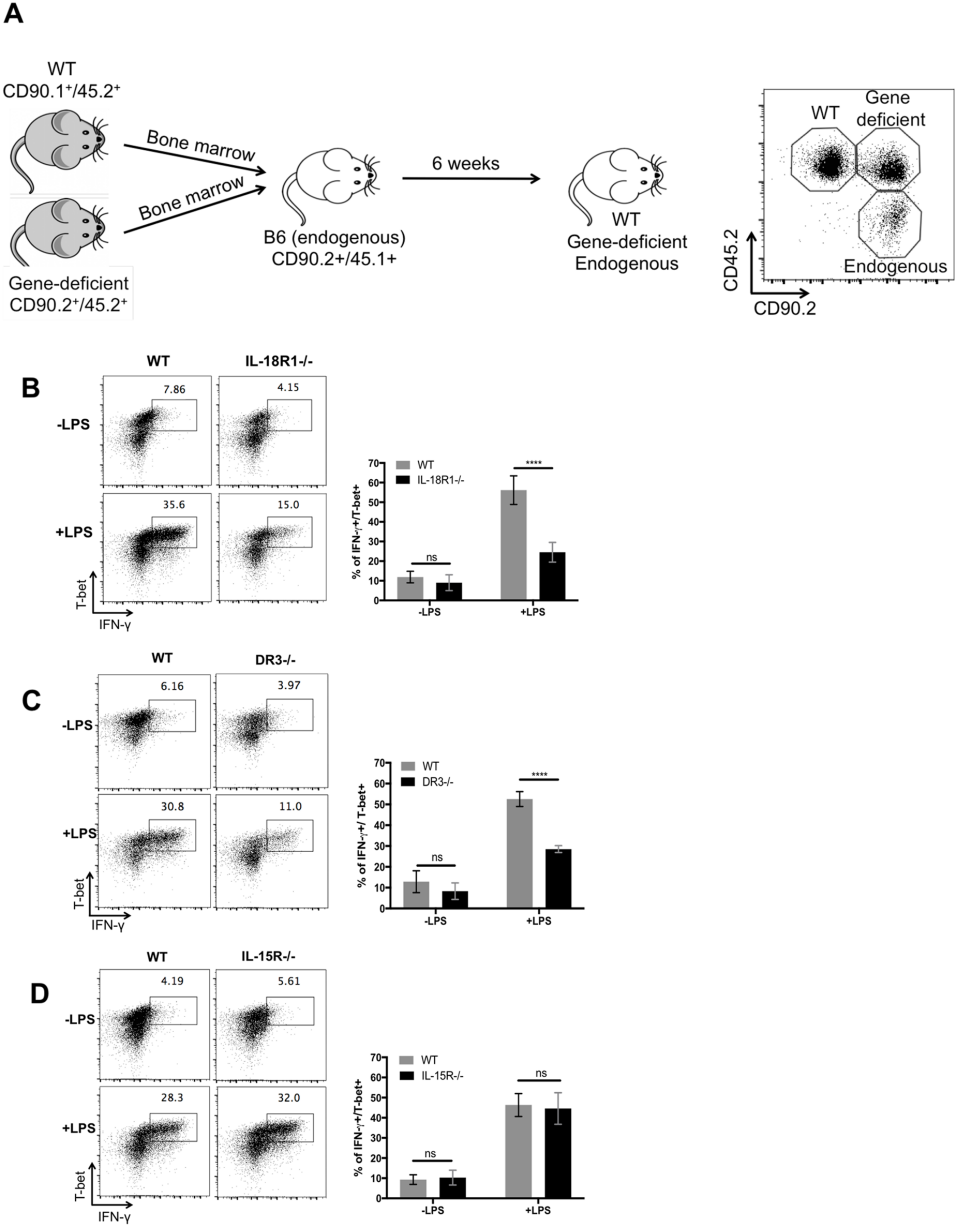
IL-18R and DR3 are required for optimal IFN-γ production. BM chimeras were infected with 5×10^5^ *Salmonella* Typhimurium followed by LPS injection two weeks after that. Spleens were collected 4 hours later to determine IFN-γ production by T-bet+ cells. (A) CD45.1+ recipients were irradiated and reconstituted with a mixture of bone marrow (BM) from wild-type (WT) (CD90.1+CD45.2+) and genetically deficient donors (CD90.2+CD45.2+). After immune reconstitution, WT and gene-deficient cells were distinguished within BM chimeras by congenic markers CD45.2 and CD90.2 as shown in representative flow plot. (B) Representative flow cytometry plots and a graph of combined data are shown for IL-18R-deficient- (B), or DR3-deficient- (C), or IL-15R-deficient (D) chimeras. Data shown were combined from two experiments containing at least three mice per group. Statistical analyses were performed by two-way ANOVA with a Bonferroni posttest. Error bars represent the mean ±SEM. p < 0.0001 (****), 0.0002 (***), 0.0021 (**), 0.0332 (*), 0.1234 (ns).

Given the increased production of IL-18, TL1A, and IL-15, in *Salmonella*-infected mice after LPS stimulation, and the known role of these cytokines in stimulating human memory T cells [34], we examined whether Th1 cells require expression of IL-18R, DR3, or IL-15R to respond to non-cognate stimulation induced by LPS. CD45.1 congenic recipient mice (CD90.2^+^ CD45.1^+^) were irradiated and reconstituted with a 1:1 mixture of WT (CD90.1^+^ CD45.2^+^) and receptor-deficient (CD90.2^+^ CD45.2^+^) bone marrow. Following immune reconstitution, three different CD4 T cell populations could be distinguished by staining for these congenic markers (Fig 4A). This experimental set-up allowed a direct comparison of WT and gene-deficient Th1 cells within a single *Salmonella*-infected host. As expected from previous reports [19,20], Th1 cells lacking IL-18R expression had a significant impairment in IFN-γ production after LPS stimulation, compared to WT Th1 cells in the same mouse (Fig 4B). Interestingly, DR3-deficient Th1 cells displayed a similar deficiency in IFN-γ production following non-cognate stimulation (Fig 4C). However, IL-15R-deficient Th1 cells had no noticeable deficiency in IFN-γ production when compared to WT Th1 cells in the same host (Fig 4D), demonstrating that the IL-15R is not essential for non-cognate activation of Th1 cells. Thus, Th1 cells in *Salmonella*-infected mice require the expression of IL-18R and DR3, but not IL-15R, to generate an optimal response to non-cognate stimuli.

### Th1 cells generated by immunization respond poorly to non-cognate stimulation

**Fig 5:**
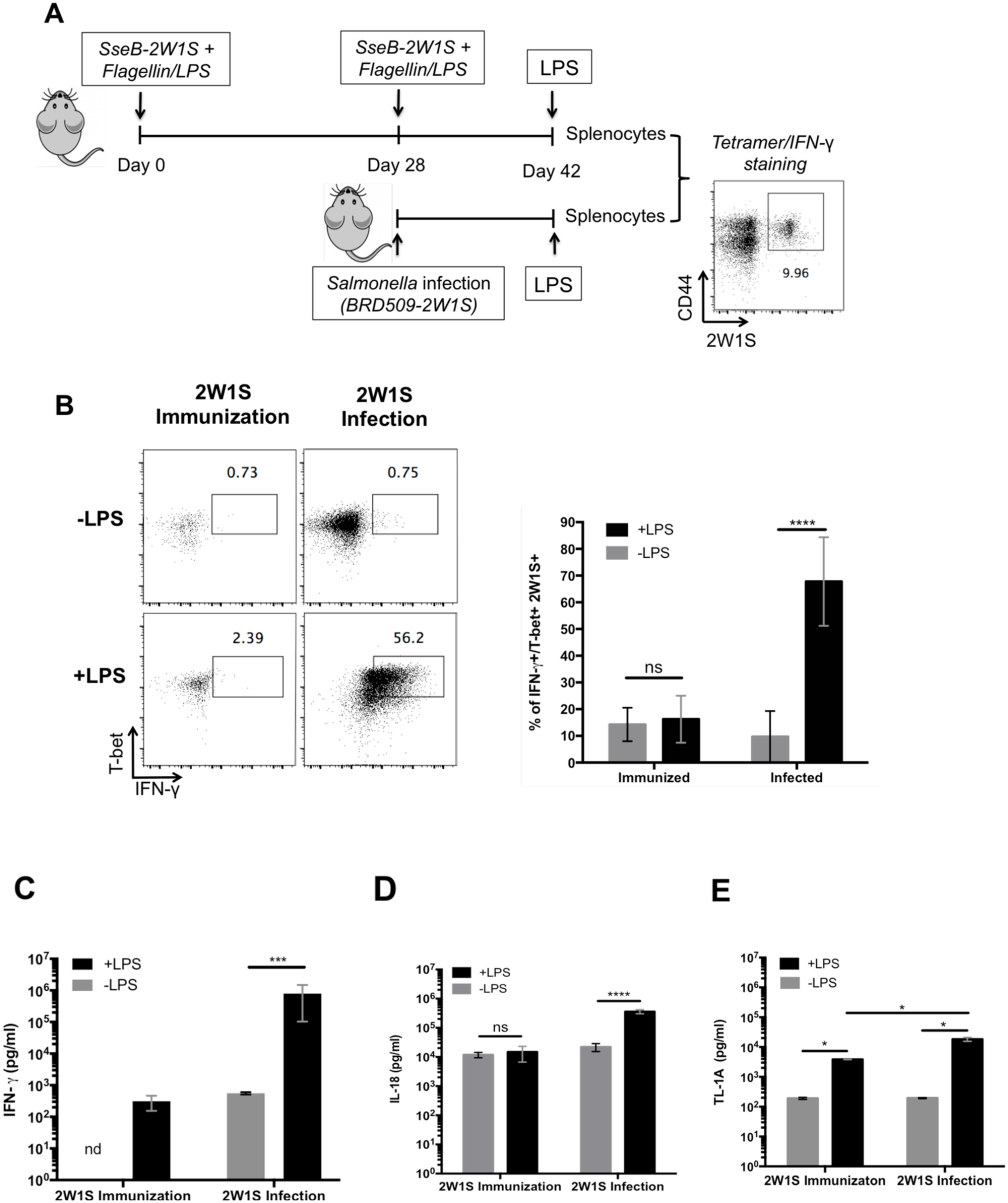
Th1 cells in immunized mice do not respond to non-cognate stimulation. (A) C57BL/6 mice were immunized intravenously with a mixture 100ug of SseB-2W1S, 100ug flagellin and 10ug LPS twice at day 0 and day 28. As a positive control, C57BL/6 mice were infected i.v. with 5×10^5^ *Salmonella* Typhimurium *(BRD509-2W1S) at day 28*. Two weeks later, immunized and infected mice were injected i.v. with 10ug LPS. Spleens and serum were harvested 4 hours later to examine IFN-γ production of 2W1S-specific T cells by tetramer and IFN-γ/T-bet staining or ELISA. (B) Representative flow cytometry plots and a graph of combined data are shown for the percentage of 2W1S-specific Th1 cells producing IFN-γ in response to LPS. (C-E) Serum was collected 4 hours after LPS injection for determining cytokine concentration by ELISA. Graphs show concentration of serum IFN-γ (C), IL-18 (D), and TL1A (E). Data shown were combined from two experiments containing at least three mice per group. Statistical analyses were performed by two-way ANOVA with a Bonferroni posttest. Error bars represent the mean ±SEM. p < 0.0001 (****), 0.0002 (***), 0.0021 (**), 0.0332 (*), 0.1234 (ns).

It is unclear whether all Th1 cells have an intrinsic capability to respond to non-cognate stimuli or whether Th1 cells generated during intracellular infection uniquely acquire this capability. Thus, we developed a model that allows for analysis of Th1 cells expanded in response *Salmonella* infection or to sub-unit vaccination. We previously generated attenuated *Salmonella* expressing a 2W1S epitope that allows direct tracking of 2W1S-specific CD4 T cells using an MHC class-II tetramer [40,41]. To develop a non-living immunogen containing the 2W1S epitope we created a fusion protein containing the protective *Salmonella* antigen SseB [42,43], fused to the same 2W1S epitope [44]. Groups of C57BL/6 mice were immunized with SseB-2W1S plus adjuvant, or were infected with *Salmonella*-2W1S, and non-cognate T cell responses of these antigen-specific T cells assessed 14 days later (Fig 5A). As expected, a large population of 2W1S-specific Th1 cells had expanded in *Salmonella*-2W1S infected mice and these cells rapidly produced IFN-γ in response to non-cognate stimulation (Fig 5B). In contrast, a smaller population of 2W1S-specific Th1 cells was generated by SseB-2W1S immunization and this population failed to respond to LPS stimulation (Fig 5B). Consistent with these data, SseB-immunized mice had reduced serum IFN-γ compared to *Salmonella*-infected mice (Fig 5C). Similarly, SseB-immunized mice had lower serum IL-18 and TL1A levels compared to *Salmonella*-infected mice (Fig 5D and 5E). These data demonstrate that Th1 cells responding to immunization have lower capacity for non-cognate activity, however, this deficiency could be due to the very different environment in infected mice where serum cytokines are significantly elevated.

### Th1 cells responding to immunization display low non-cognate responses in an infected host

**Fig 6:**
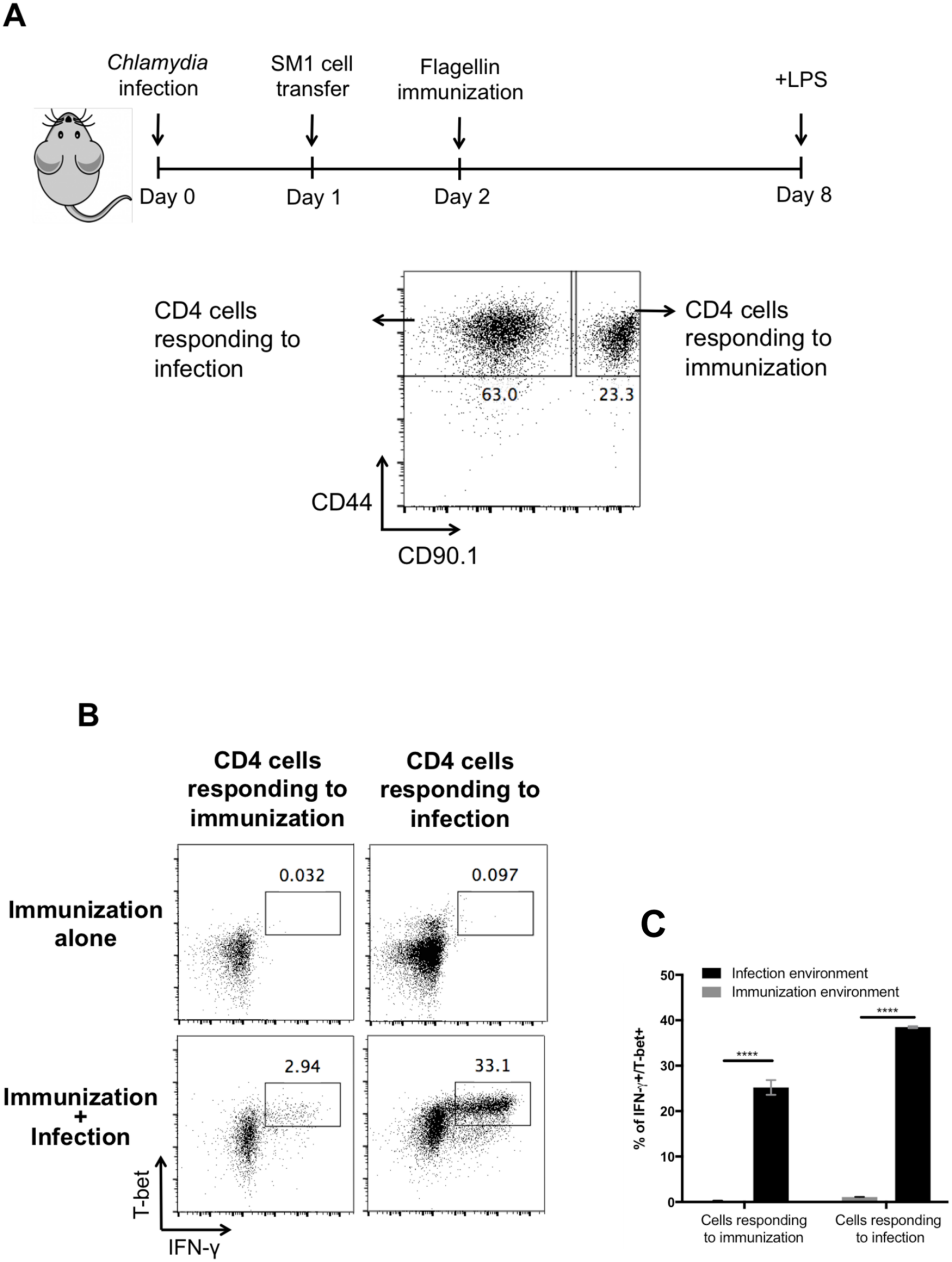
Th1 cells generated by immunization respond weakly in infected environment. (A) C57BL/6 mice were infected i.v. with 1×10^7^ *Chlamydia muridarum*. On the following day, CD90.1 SM1 cells were transferred adoptively into infected or uninfected mice at the dose of 0.5×10^6^ cells/mouse. Flagellin immunization was performed intravenously one day after SM1 cell transfer, 1ug flagellin/mouse. At day 8 postinfection, 10ug LPS was administered i.v., and spleens were collected 4 hours later to determine IFN-γ production by SM1 cells (CD90.1+) and endogenous CD4 T cells (CD90.2) by flow cytometry. (B) Representative flow cytometry plots and (C) a graph of combined data are shown. Data shown were combined from two experiments containing at least three mice per group. Statistical analyses were performed by two-way ANOVA with a Bonferroni posttest. Error bars represent the mean ±SEM. p < 0.0001 (****), 0.0002 (***), 0.0021 (**), 0.0332 (*), 0.1234 (ns).

Given the difference in serum cytokines, it was of interest to directly compare Th1 cells responding to infection or immunization in the same inflammatory environment. An experimental setup was generated where two Th1 cell populations from different responses could be stimulated in exactly the same host. Transferred Th1 cells responding to peptide immunization were visualized using CD90.1^+^ *Salmonella*-specific SM1 TCR transgenic CD4 T cells (SM1) [45], while endogenous CD90.2^+^ CD4 T cells responding to *Chlamydia* infection were monitored simultaneously. CD90.1^+^ SM1 T cells were adoptively transferred into CD90.2^+^ C57BL/6 recipient mice infected with *Chlamydia* and then expanded by flagellin peptide immunization (Fig 6A). In agreement with the experiments above, expanded SM1 cells in peptide-immunized mice were unable to produce IFN-γ (Fig 6B-top) while 30–40% of endogenous Th1 cells in *Chlamydia*-infected mice produced IFN-γ to non-cognate stimulation (Fig 6B-bottom and 6C). In marked contrast, only a small proportion of expanded SM1 Th1 cells were able to produce IFN-γ in the same *Chlamydi*a-infected animal (Fig 6B-bottom). However, it should be noted that this small population of IFN-γ producers still represents around 20% of all T-bet^+^ SM1 cells (Fig 6C). Thus, Th1 cells responding to immunization appear to have some capacity for non-cognate responsiveness but at a significantly reduced level when directly compared to Th1 cells responding to infection.

### Th1 cells responding to infection or immunization express similar levels of IL-18R and DR3

**Fig 7:**
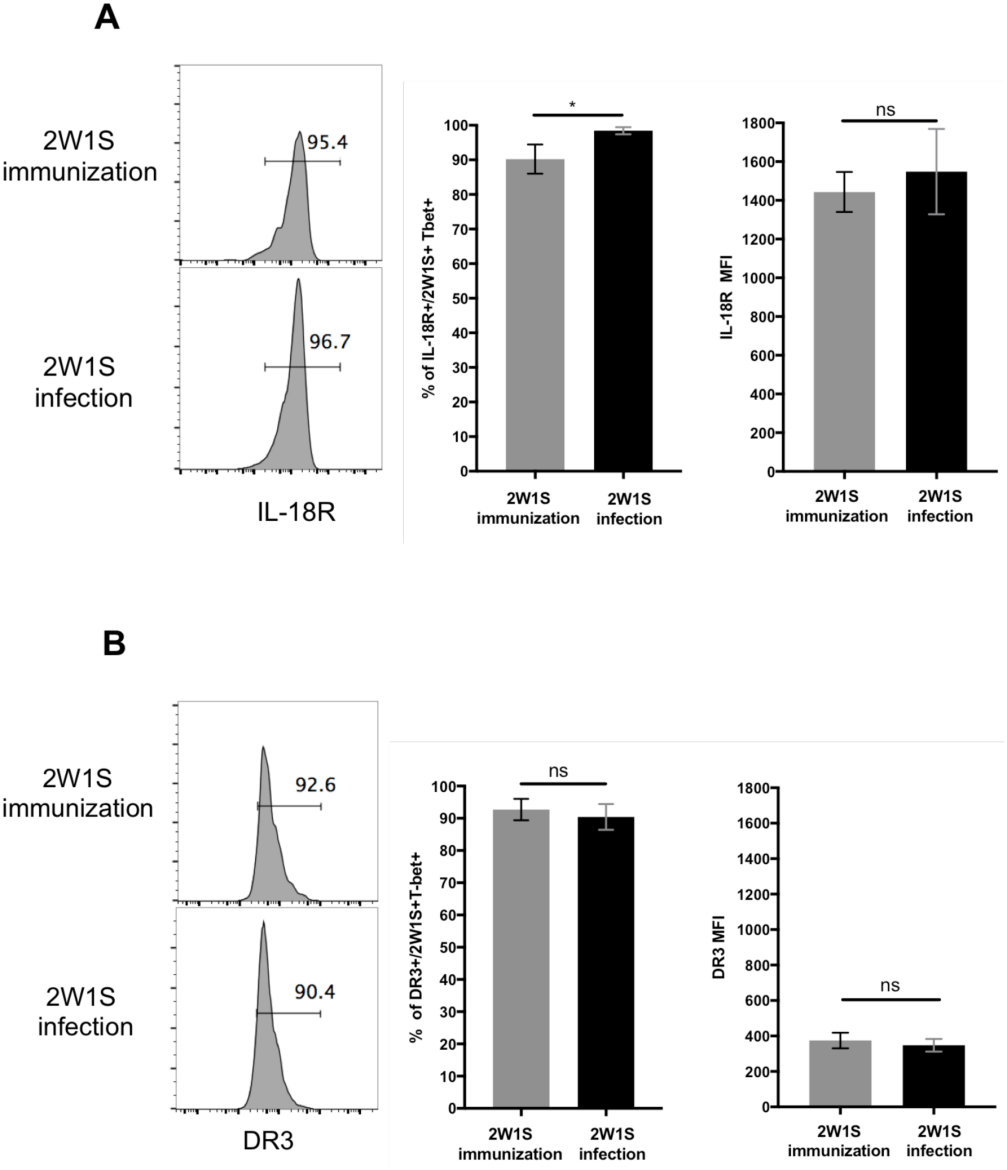
IL-18 and DR3 expression on Th1 cells. C57BL/6 mice were immunized intravenously with a mixture 100ug of SseB-2W1S, 100ug flagellin and 10ug LPS twice at day 0 and day 28. As a positive control, C57BL/6 mice were infected i.v. with 5×10^5^ *Salmonella* Typhimurium *(BRD509-2W1S) at day 28*. Two weeks later, spleens were harvested to examine surface marker expression on 2W1S-specific T-bet^+^ cells. Representative flow cytometry plots and graphs of combined data are shown for of IL-18R (A) and DR3 (B) expression. Data shown were combined from two experiments containing at least three mice per group. Statistical analyses were performed by two-way ANOVA with a Bonferroni posttest. Error bars represent the mean ±SEM.

Given the lower capacity for non-cognate responses by Th1 cells generated by immunization, it was of interest to determine whether this was due to differential expression of IL-18R and DR3. However, almost all expanded 2W1S-specific CD4 Th1 cells in both *Salmonella*-infected and SseB-2W-immunized mice expressed IL-18R and DR3, although the proportion of IL-18R^+^ Th1 cells was somewhat higher in *Salmonella*-infected mice (Fig 7A and 7B). Additionally, the median fluorescence intensity (MFI) of IL-18R and DR3 expression was comparable on Th1 cells generated in both groups of mice (Fig 7). Thus, these results suggest that Th1 cells developing in response to immunization are broadly similar to Th1 cell in infected mice with respect to IL-18 and TL1A responsiveness and therefore some unknown additional signal determines differential responsiveness.

### Mice with T cells lacking non-cognate stimulatory capacity are more susceptible to multiple infections

**Fig 8:**
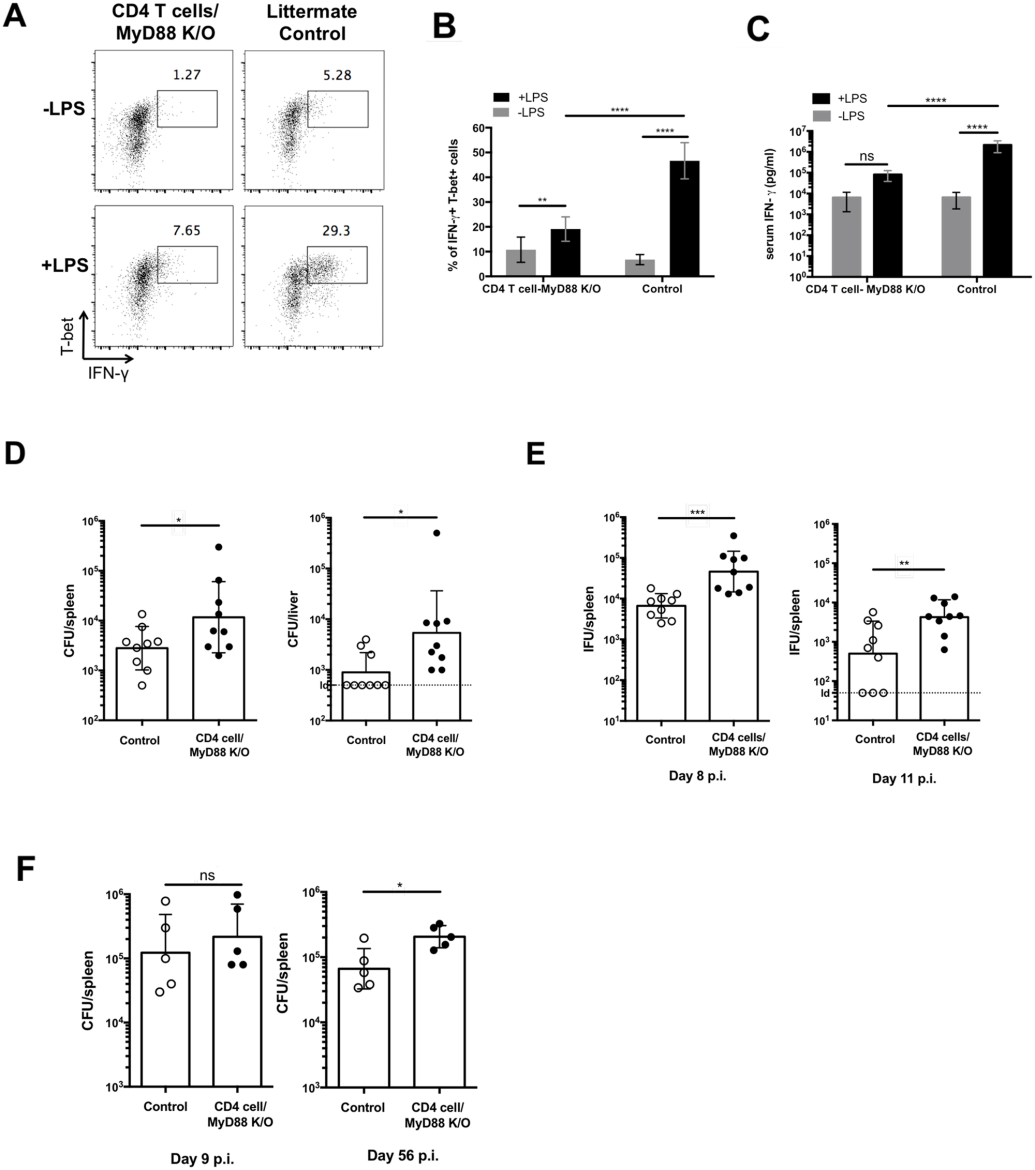
Mice lacking Myd88 in CD4 T cells are susceptible to multiple infections. CD4-Cre Myd88^fl/fl^ mice and littermate control were infected with 5×10^5^ *Salmonella* Typhimurium. Two weeks later, infected mice were injected i.v. with 10ug LPS. (A-C) Spleens were collected 4 hours later to examine IFN-γ production by Th1 cells by flow cytometry. Representative flow cytometry plots (A) and a graph of combined data (B) are shown. Serum was also collected 4 hours after LPS treatment to determine IFN-γ concentration by ELISA (C). (D-F) Bacterial loads in the spleen and/or liver of individual mice were determined at day 35 post-infection for *Salmonella*-infected mice (D), day 8 and day 11 for *Chlamydia*-infected mice (inclusion-forming units, IFC) (E), or day 9 (early stage) and day 56 (late stage) for *Brucella*-infected mice (F). Data shown were combined from two experiments containing at three to five mice per group. Statistical analyses were performed by two-way ANOVA with a Bonferroni posttest. Error bars represent the mean ±SEM. p < 0.0001 (****), 0.0002 (***), 0.0021 (**), 0.0332 (*), 0.1234 (ns).

Finally, to evaluate the *in vivo* relevance of non-cognate responses of Th1 cells, we generated *Myd88*^*fl/fl*^ *Cd4-cre* mice containing T cells lacking the adaptor protein Myd88, a critical mediator of IL-18R signaling [46]. As expected, littermate control mice infected with *Salmonella* developed a robust Th1 cell IFN-γ response to non-cognate stimulation (Fig 8A and 8B). In contrast, Th1 cells in *Salmonella*-infected CD4-MyD88-deficient mice displayed a markedly lower IFN-γ response to LPS injection (Fig 8A and 8B). Consistent with these data, serum IFN-γ in *Myd88*^*fl/fl*^ *Cd4-cre* mice was significantly lower than in littermate control mice after LPS stimulation (Fig 8C).

Next, we examined the ability of CD4-MyD88-deficient mice to eliminate intracellular bacterial infections. Following *Salmonella* infection, *Myd88*^*fl/fl*^ *Cd4-cre* mice had significant higher bacterial counts in both the liver and spleen (Fig 8D), indicating a reduced capacity to eliminate bacteria. It is important to note that some *Myd88*^*fl/fl*^ *Cd4-cre* mice are able to control infection, possibly as a result of DR3 signaling or cognate interactions to produce IFN-γ. As with *Salmonella* infection, clearance of *Chlamydia* was significantly reduced in *Myd88*^*fl/fl*^ *Cd4-cre* mice compared to littermate controls (Fig 8E). Finally, *Myd88*^*fl/fl*^ *Cd4-cre* mice were also found to have significantly higher bacterial burdens during late stage (day 56) clearance of *Brucella* infection (Fig 8F). Thus, mice containing T cells with lower capacity for non-cognate responses have reduced ability to clear multiple intracellular pathogens. Together these data point to an important role for non-cognate responses in amplifying Th1 cell protective capacity in vivo.

## Discussion

Macrophage activation is a critical host defense mechanism against a wide range of intracellular pathogens [47]. IFN-γ produced by CD4 Th1 cells amplifies the killing capacity of infected tissue macrophages, allowing them to restrain intra-phagosomal replication of bacteria [4]. This bacterial killing process within the macrophage is antigen-non-specific and likely involves increased production of reactive oxygen and nitrogen species and the restriction of nutrient availability in the phagosome [48,49]. Effector Th1 cells can be activated efficiently by infected tissue macrophages that display peptide/MHC on the cell surface [4]. Indeed, in a mouse model of *M*. *tuberculosis* infection, MHC class-II-deficient macrophages were less able to restrict bacterial growth than MHC class-II-expressing macrophages [50]. However, other studies have shown that intra-macrophage pathogens can lower MHC class-II expression and antigen processing as a means to evade detection by the adaptive immune system [51–56]. Thus, relying exclusively on cognate antigen recognition in an infected tissue may leave a host vulnerable to infection with intracellular bacteria [57].

Although the initial activation and expansion of antigen-specific CD4 T cells is strictly controlled by TCR recognition of cognate antigen, effector T cells can also be stimulated to secrete cytokines in the absence of TCR ligation [9]. Th1, Th2, and Th17 cells can each be activated in the presence of cytokines, and in particular, IL-1 family members IL-18, IL-33, and IL-1β, appear to play a key role in regulating this process [17]. Our data show that mice lacking T cell expression of the key adaptor molecule Myd88 display a small but significant deficiency in the clearance of *Salmonella*, *Chlamydia*, and *Brucella*. This deficiency correlated with reduced non-cognate activity from Th1 cells and we have previously shown that CD4 T cells lacking TLR4 expression are fully capable of responding to LPS-induced inflammation. Thus, non-cognate stimulation of Th1 cells likely plays a key role in protection against multiple intracellular bacterial pathogens. This finding fits well with prior studies showing that depletion or deficiency in IL-18 results in a reduced capacity to resolve intracellular bacterial infections [19,58–60]. Similarly, recent data show that MyD88 expression in CD4 T cells is important for the resolution of *Chlamydia* infection from the genital tract independently of TLR or IL-1R [61]. While the effect of T cell Myd88-deficiency is relatively modest in our study (around 1 log), it is important to remember that these mice still have a large pool of expanded Th1 cells that are able to respond to cognate signals or non-cognate activity mediated by other cytokines. Furthermore, even CD4 depletion does completely eliminate *Salmonella* immunity since B cells and CD8 T cells also participate in bacterial control [62,63]. Precisely determining the individual contribution of the cognate response is made difficult by the fact that few major *Salmonella* epitopes are known, TCR stimulation is required for initial Th1 formation, and expanded Th1 cells survive poorly after transfer to MHC class-II-deficient recipients [19,64]. However, our data support the idea that non-cognate T cell stimulation plays an important contribution in the clearance of several intracellular bacterial infections.

Aside from IL-1 family members, a wide range of cytokines are known to elicit non-cognate responses from effector T cells [16]. Importantly, IL-12, IL-15, IL-18, and TL1A have recently been shown co-operate to elicit IFN-γ production from human CD4 memory cells [34]. Our microarray analysis showed that TL1A expression was highly increased after LPS-induced inflammation and our serum cytokine measurements confirmed that TL1A, IL-15, and IL-18 levels all increased. Interestingly, the regulation of TL1A differed somewhat from IL-15 and IL-18 since serum levels of TL1A increased in both uninfected and infected mice after LPS injection. In a previous study, IL-15 synergized with TL1A to induce TCR-independent cytokine production from human mucosal memory CD4 T cell that co-express IL-18Rα and DR3 [34]. However, our mixed bone marrow chimera experiments show that IL-18Rα and DR3, but not IL-15R are essential for eliciting IFN-γ production of Th1 cells during *Salmonella* infection. Thus, our data show an important requirement for IL-18 and TL1A in non-cognate activation of CD4 T cells but suggest that IL-15 is not required. It seems possible that IL-15 plays a prominent role in stimulating human CD4 T cells or mucosal CD4 T cells but is dispensable in the murine *Salmonella* model, perhaps because of the contribution of additional cytokines such as IL-33 [20]. Given the residual IFN-γ production present in both DR3-, and IL-18-deficient CD4 T cells, it is also possible that additional cytokines can drive this response. Experiments examining DR3/IL-18R-deficient CD4 T cells will be needed to resolve this issue.

Our data show that Th1 cells generated by SseB immunization have similar IL-18R and DR3 expression when compared with Th1 cells responding to *Salmonella* infection. However, Th1 cells generated by immunization respond poorly to LPS stimulation. This deficiency appears to be T cell intrinsic since Th1 cells generated by immunization have weaker non-cognate responses than Th1 cells responding to infection in the same environment. This has important implications for the development of subunit vaccines against intracellular pathogens since this approach might induce Th1 cells with reduced functional capacity compared to live attenuated vaccines. It is notable that CD4 cells responding to immunization displayed a lower percentage of T-bet^+^ cells than CD4 cells responding to infection, suggesting differential strength of TCR stimulus and/or signals delivered by DCs might lead to poor Th1 commitment. Several different immunization protocols were explored before using SseB and flagellin immunization, and most gave a low percentage of T-bet+ cells that prevented a direct comparison to Th1 cells generated by infection. A more detailed comparison of Th1 cell populations generated by infection or immunization is called for since this issue may be vitally important for the design of vaccines against intracellular pathogens.

Together, our data show that non-cognate activation of Th1 cells plays an important role in host defense against multiple intracellular pathogens and indicates that IL-18 and TL1A cooperate to maximize T cell mediated killing in infected tissues. These experiments also suggest that Th1 cells generated by immunization may have poorer non-cognate capability than an equivalent population generated by live infection. Greater understanding of this process could lead to better strategies to control persistent infections, particularly intracellular pathogens that can regulate macrophage MHC class-II expression.

## Materials and methods

### Ethics statement

This study was carried out in strict accordance with the recommendations in the Guide for the Care and Use of Laboratory Animals of the National Institutes of Health. The University of California Davis is accredited by the Association for Assessment and Accreditation of Laboratory Animal Care (AAALAC). All animal experiments were approved by University of California Davis Institutional Animal Care and Use Committee (IACUC) (Protocol number 18299 and 18677).

### Mice

C57BL/6, B6.SJL- Ptprc^a^Pepcb/BoyJ, B6.PL-Thy1a/CyJ, *Cd4*-cre, Myd88^fl/fl^, IL-18R-deficient, IL-15R-deficient mice were purchased from The Jackson Laboratory at 6–8 weeks of age. DR3-deficient mice [65], were maintained at the University of Southampton. T cell-specific MyD88-deficient mice were generated for this study by intercrossing *Cd4*-cre mice and Myd88^fl/fl^ mice. All mice were genotyped before use by PCR according to protocols provided by The Jackson Laboratory. CD90.1 RAG-deficient SM1 TCR transgenic mice were bred at UC Davis and assessed by flow cytometry [45,66].

### Bacterial strains, infection, and bacterial load determination

*S*. Typhimurium AroA^-^ (BRD509) was originally provided by Dr. D. Xu, (University of Glasgow, Glasgow, U.K). A *S*. Typhimurium BRD509 strain expressing the 2W1S epitope (EAWGALANWAVDSA) (strain SPN555 [41]) was generated by P22 transduction using a wild-type donor strain expressing 2W1S provided by Dr. M. Jenkins, University of Minnesota, Minneapolis, MN. *Salmonella* strains were grown overnight in Luria-Bertani broth without shaking and diluted in PBS after estimation of bacterial concentration by a spectrophotometer at OD600. Mice were infected intravenously (i.v.) with 5×10^5^ bacteria unless otherwise indicated and the actual bacterial dose administered was confirmed by plating serial dilutions onto MacConkey agar plates. To determine bacterial colonization in vivo, spleens and livers from infected mice were collected in PBS on ice and homogenized. Samples were mixed thoroughly and serial dilutions were plated onto MacConkey agar plates. After overnight incubation at 37°C, bacterial plates were counted and bacterial burdens calculated for each individual organ. *Chlamydia muridarum* strain Nigg II was purchased from ATCC and cultured in HeLa 229 cells (also obtained by ATCC) in Dulbecco’s modified Eagle’s medium supplemented with 10% fetal bovine serum. Elementary bodies (EBs) were purified by discontinuous density gradient centrifugation as previously described and stored at 80°C [67]. The number of Inclusion-Forming Units (IFUs) was determined by in vitro infection of HeLa 229 cells and enumeration of inclusions stained with anti-*Chlamydia* MOMP. C57BL/6 mice were infected i.v. with 1×10^7^ *C*. *muridarum* and bacterial burdens determined as previously described [68]. *B*. *abortus* strain 2308 was cultured on blood agar plates for preparation of mouse inocula and suspended in physiologic saline. Mice were inoculated intraperitoneally (i.p.) at a total dose of approximately 5x10^4^ CFU per mouse. Groups of five mice were sacrificed at day 56 post-infection, and bacterial colonization of spleens was quantified by plating serial dilutions of tissue homogenates on Tryptic Soy Agar. All experiments with *Brucella abortus* were performed under Biosafety Level 3 containment.

### Bone marrow chimeras

Mixed BM chimeras were generated by transferring bone marrow from CD45.2^+^ congenic donors into CD45.1^+^ congenic recipients. CD45.1^+^ congenic mice (B6.SJL) were irradiated twice on consecutive days using 625 rads from cesium source before receiving bone marrow from wild-type and gene-deficient donors. Bone marrow was collected from the femurs and tibias of CD90.1^+^ wild-type mice (B6. PL) and CD90.2^+^ gene-deficient mice. Cells were counted using a hemocytometer and combined at a 1:1 ratio before i.v. injection into irradiated recipients. Following BM transfer, chimeras were maintained on Polymyxin B Sulphate (13mg/L) and Neomycin Sulphate (38mg/L) for at least 10 days, and blood collected 4 weeks later to confirm chimerism by flow cytometry using CD90.1 and CD90.2 antibodies. Mixed bone-marrow chimeras were used 6–8 weeks following BM transfer and 2 weeks after the withdrawal antibiotics.

### Adoptive transfer of TCR transgenic T cells

Spleens from SM1 trangenic mice (CD90.1^+^, RAG-deficient) were collected and homogenized in PBS. SM1 cells were transferred intravenously (i.v.) into C57BL/6 recipient mice (CD90.2^+^) at a dose of 0.5×10^6^ cells/mouse. After adoptive transfer, SM1 cells were distinguished from endogenous cells by flow cytometry after staining with antibodies specific to CD4 and CD90.1.

### In vivo stimulation with LPS

Ultrapure LPS from E. coli strain EH100Ra (Alexis, TLRgrade) was diluted in 1X PBS (UltraPure PBS, GIBCO) and injected intravenously to mice at a dose of 10μg/mouse. Spleens were harvested from infected or uninfected mice 4 hours after the administration of LPS and processed for flow cytometric analysis of cytokine production.

### Flow cytometry

Spleens were homogenized in PBS supplemented with 2% FBS. A single-cell suspension was prepared at the concentration of 5×10^7^ cells/ml and stained with various fluorophore-conjugated antibodies (eBioscience, or Tonbo Biosciences) in Fc block (culture supernatant from the 24G2 hybridoma, 2% mouse serum, 2% rat serum, and 0.01% sodium azide) for 30–60 min on ice. Cells were permeabilized and fixed overnight using Foxp3 intra-nuclear staining kit (eBioscience) before they were ready for intracellular cytokine or transcription factor staining. Stained cells were analyzed with Fortessa (BD) with appropriate compensation controls. Flow cytometry data were analyzed with FlowJo software (TreeStar).

### Microarray analysis

Tissue samples (spleens) were collected from *Salmonella*-infected or *Chlamydia*-infected mice that were untreated or stimulated i.v. with 10μg *E*. *coli* LPS. Spleens were promptly snap frozen by submersion of cryovials in liquid nitrogen and stored at -80°C until RNA extraction. Total RNA was extracted according to the manufacturer’s instructions using TRIzol reagent (Ambion) and quantified using a Nanodrop spectrophotometer (Thermo Scientific). RNA was DNase-treated using a DNA-free kit (Ambion) and assessed for quality based on agarose gel electrophoresis and OD260/280. Total RNA was submitted to the UC Davis Microarray Core Facility for RNA processing, hybridization and scanning. Briefly, cDNA was synthesized from total RNA and biotin labeled using the Ambion WT (Life Technologies) and GeneChip WT Terminal Labeling (Affymetrix) kits, according to the respective manufacturers' instructions. Biotinylated cDNA was hybridized to the GeneChip Mouse Gene 1.0 ST Array (Affymetrix) in the GeneChip Hybridization Oven 640 overnight, and GeneChips washed and stained in the Affymetrix Fluidics Station 450. Probe intensities were measured using a GeneChip Scanner 3000 7G (Affymetrix). Data was processed directly from CEL files using dchip (DNA-Chip Analyzer, www.dchip.org) with standard settings. Briefly, as detailed in the dchip User's Manual and related articles [69,70], arrays were normalized at probe cell level for comparison and expression values were calculated with standard errors, then averaged for each group. Two-group comparisons were made by paired t-test between mean group expression values, with 90% confidence intervals of fold changes computed. Data were filtered to include only comparisons with p values greater than 0.05. Datasets were sorted, compared, and graphed after import into Microsoft Excel. The array data discussed in this publication have been deposited in NCBI's Gene Expression Omnibus and are accessible through GEO Series accession number GSE95731 (https://www.ncbi.nlm.nih.gov/geo/query/acc.cgi?acc=GSE95731).

### Sseb-2W1S protein purification and immunization

*Salmonella* antigen, SseB, was cloned from *Salmonella* genomic DNA and inserted into the His-tag pRSET vector and overexpressed in *E*.*coli* BL21star DE3 cells (ThermoFisher Scientific). To generate an SseB protein expressing the 2W1S epitope (EAWGALANWAVDSA) (SseB-2W), the 2W1S sequence was cloned in-frame onto the C-terminus of SseB and expressed in the same *E*. *coli* expression system. Recombinant *E*. *coli* strains expressing SseB-2W1S protein were cultured overnight in Luria-Bertani (LB) broth in 1mM IPTG at 28°C. Bacteria culture was centrifuged then pellet resuspended in Bugbuster (EMD Millipore) and inclusion bodies resuspended in 8M Urea denaturing buffers. Recombinant SseB-2W1S was purified using Ni-NTA His-Bind resin (EMD Millipore) according to the manufacturer’s protocol before dialysis in 1X PBS to remove traces of urea. Samples were subsequently concentrated using 10,000 MWCO Amicon tubes (EMD Millipore) and protein concentration was determined using BCA method (Thermo Fisher Scientific). C57BL/6 mice were immunized intravenously with a mixture 100ug of SseB-2W1S, 100ug flagellin and 10ug LPS twice at day 0 and day 28.

### Flagellin purification

LPS-deficient *Salmonella* Typhimurium χ4700 was cultured overnight at 37°C, no shaking. Bacteria culture was centrifuged, then pellet was resuspended in PBS/HCl at pH2 for 30 min at room temperature. Solution then was spun down and supernatant collected to harvest flagellin by ultracentrigation and ammonium sulfate precipitation. Polymeric flagellin was heated at 70°C for 1 hour to form monomeric flagellin. Trace remaining LPS was removed ultilizing detoxigel columns (Thermo Fisher Scientific).

### Tetramer staining and enrichment of 2W1S-specific CD4 T cells

Spleens were collected, homogenized in FACS buffer (PBS with 2% FBS), and stained with PE-conjugated 2W1S:I-A^b^ tetramer in the presence of Fc block for an hour at room temperature in dark. Cells were then washed and incubated with anti-PE microbeads for 30 min on ice before being enriched via LS MACS colums (Miltenyi Biotec) to collect tetramer-positive cells (bound fraction). Bound fraction was collected and ready for extracellular and intracellular stainings. Stained samples were analyzed with Fortessa (BD) and FlowJo software (TreeStar).

### Serum cytokine ELISAs

Blood was collected from thoracic cavity of euthanized mice and placed on ice to allow clotting. Samples then were centrifuged to separate serum and clot. Serum was harvested and stored at -20°C. Cytokine ELISA for IFN-γ and IL-15/IL-15R were performed using Ready-Set-Go kit (eBioscience) with provided instruction, and concentrations were determined by the protein standard provided. IL-18 ELISA was conducted following the same protocol with capture antibody, detection antibody, and recombinant IL-18 as a standard purchased from Medical and Biological Labs (MBL). TL1A (TNFSF15) ELISA was performed with CUSABIO ELISA kit purchased from Flarebio Biotech LLC. After substrate was added, plates were read at 450 nm with a microplate reader (Spectra Max M2, Molecular Devices) and analyzed in Microsoft Excel.

### Statistical analyses

All statistical analyses were performed as described in the figure legends with GraphPad Prism version 6. All error bars represent the mean ± SEM.
